# Robust ammonia oxidation by “*Candidatus* Nitrosacidococcus tergens” across a broad pH range

**DOI:** 10.1128/mbio.02975-25

**Published:** 2026-04-24

**Authors:** Ida F. Peterse, Claudia Frey, Reinier A. Egas, Guylaine H. L. Nuijten, Annelies J. Veraart, Sebastian Lücker

**Affiliations:** 1 Department of Microbiology, Radboud Institute for Biological and Environmental Sciences, Faculty of Science, Radboud University 98810 , Nijmegen, the Netherlands; 2 Department of Ecology, Radboud Institute for Biological and Environmental Sciences, Faculty of Science, Radboud University 98810 , Nijmegen, the Netherlands; 3 Department of Environmental Science, University of Basel 27209, Basel, Switzerland; Georgia Institute of Technology, Atlanta, Georgia, USA

**Keywords:** ammonia oxidation, proton stress, Nitrosacidococcus, nitrogen dynamics, nitrogen stable isotopes

## Abstract

Acidophilic ammonia-oxidizing bacteria (AOB) have only recently been discovered. These organisms hold great promise for acidic wastewater treatment; however, their physiology remains poorly understood compared to that of neutrophilic AOB. Here, we investigated the physiology of the acidophilic AOB *“Candidatus* Nitrosacidococcus tergens*”* sp. RJ19 across a broad pH range (2.5–7.0) using a specialized bioreactor system. We monitored nitrogen (N) transformations combined with microbial community composition and transcriptomic profiles, focusing on nitrogen metabolism and proton stress responses. Our results show that above pH 6.0, “*Ca*. Na. tergens*”* performs complete and stoichiometric conversion of ammonium to nitrite, coinciding with isotopic fractionation effects specific for ammonia oxidation and increased expression of key ammonia oxidation genes. The apparent absence of *nirK* and *cycA* did not impede ammonia oxidation, suggesting that these genes are non-essential in this context. Below pH 6.0, nitric oxide and nitrate accumulated, and nitrous oxide (N₂O) levels, although negligible compared to the other N-compounds, peaked near pH 4.0. Stable isotope analysis, including the site-specific ^15^N-enrichment at the inner (α) and outer (β) nitrogen positions of the N_2_O molecule, indicated nitrifier-denitrification as the source of N₂O, supported by the highest *norB* expression at this pH. These findings provide new insights into the acid-tolerant physiology of “*Ca*. Na. tergens*”* and advance its potential application in engineered nitrogen removal systems under acidic conditions.

**IMPORTANCE:**

The world is facing a climate crisis intensified by human-driven nutrient pollution. Ammonia and the bacteria that oxidize it are central both to the global nitrogen cycle and to wastewater treatment. The acidophilic ammonia oxidizer “*Candidatus* Nitrosacidococcus tergens” was previously shown to oxidize ammonia under highly acidic conditions; however, a complete understanding of its metabolism is lacking. Our study now shows that “*Ca*. Na. tergens” performs canonical ammonia oxidation across a broad pH range. At pH values below 6, however, a combination of chemical and biological processes leads to the production of nitrate, nitric oxide, and the greenhouse gas nitrous oxide. In addition, we show that these bacteria adapt to proton stress through mechanisms beyond transcriptional mechanisms. Our study highlights the robust metabolism of acidophilic ammonia oxidizers and expands our understanding of nitrification under acidic conditions.

## INTRODUCTION

Human activities have significantly altered nitrogen (N) loading to aquatic and terrestrial ecosystems. While this has contributed to well-known disturbances such as eutrophication in aquatic ecosystems, elevated N-load also impacts nitrogen-cycling processes such as nitrification, the biological oxidation of ammonia (NH_3_) to nitrate (NO_3_^−^), a key process in the global nitrogen cycle (1). For instance, increased ammonium (NH_4_^+^) concentrations can alter nitrification rates, potentially leading to the acidification of ecosystems. Although nitrification in acidic soils was already reported over a century ago (2), it was long assumed that the first step of nitrification, ammonia oxidation, was unlikely to occur in acidic environments. This assumption was based on the fact that the chemical equilibrium of NH_3_ and NH_4_^+^ (pK_a_ 9.25) decreases the availability of NH_3_ at low pH, which is considered the substrate for the ammonia monooxygenase (AMO) enzyme catalyzing the first oxidation step of the nitrification pathway (3).

Although no significant correlation between soil pH and nitrification rates was found (4), it has only become clear with the recent isolation of various acidophilic and acid-tolerant ammonia-oxidizing microbes (5–8) that ammonia oxidation can indeed occur under (highly) acidic conditions (for a comprehensive review, see reference 9). The first acidophilic ammonia oxidizer described was the archaeon *Nitrosotalea devaniterrea* isolated from acidic agricultural soil (pH 4.5) (7, 10). This was soon followed by the discovery of acid-tolerant and acidophilic ammonia-oxidizing bacteria (AOB), including “*Candidatus (Ca*.) Nitrosoglobus terrae” (5), “*Ca*. Nitrosacidococcus tergens” (6), and “*Ca*. Nitrosacidococcus urinae” (8), all members of the class *Gammaproteobacteria*. Although these strains show activity toward neutral pH, they all were isolated from acidic environments such as an air scrubber filtering ammonia-rich emissions from a pig stable (pH 2.9–4.3) (6), sludge of urine nitrification reactors (maintained between pH 5.8 and 7.0) (8), and soil of acidic tea fields (pH 3.0) (5).

Interestingly, especially “*Ca*. Nitrosacidococcus tergens” showed activity in an extremely broad pH range from 2.5 to 7.0 in a continuous culture with ammonium as the sole energy substrate (6). At acidic pH, apart from proton stress, challenging pH homeostasis, and low substrate availability, nitrogen species can react chemically, resulting in reactive and toxic nitrogenous compounds (11). Despite these constraints, multiple acid-tolerant ammonia oxidizers withstand these conditions, for example, by the formation of cell aggregates (5, 6). However, in-depth insights into their physiology in combination with nitrosative stress at acidic pH are still lacking.

During the first step in the canonical ammonia oxidation pathway at neutral pH, NH_3_ is oxidized to hydroxylamine (NH_2_OH), carried out by AMO, which requires oxygen (O_2_) for activation (12). Then, hydroxylamine oxidoreductase (HAO) oxidizes NH_2_OH to nitric oxide (NO) (13). Under anaerobic conditions, NH_2_OH can also be oxidized by the cytochrome P460 to nitrous oxide (N_2_O) (14). The last step of the ammonia oxidation pathway is the oxidation of NO to nitrite (NO_2_^−^). Until now, no nitric oxide oxidoreductase has been identified, although some candidates were previously proposed, for example, the red copper protein nitrosocyanin (15–17) and a NirK-type nitrite reductase working in reverse (13, 18). Although nitrosocyanin is highly upregulated during ammonia oxidation (17), it is unlikely to serve as a redox-active protein (19).

In acid-tolerant gammaproteobacterial AOB, there are some notable genomic differences. Interestingly, nitrite reductase NirK was not found in the genomes of the *Nitrosacidococcus* and the *Nitrosogolobus* species (5, 6, 8), while it is highly conserved in almost all sequenced AOB genomes to date. Moreover, *cycA*, which is present in all genome-sequenced AOB (20), seemed to be missing in acidophilic and acid-tolerant AOB genomes. *CycA* is part of the *haoAB-cycAB* operon and is expected to shuttle electrons between the HAO and cytochrome *c*_M_552 (21). Considering these genomic features, canonical ammonia oxidation appears uncertain under acidic conditions, particularly as “*Ca*. Na. tergens” at pH 3.5 produced NO and NO_3_^−^, rather than NO_2_^−^ as dominant N-compounds (6). However, this is likely a result of the chemical conversion of NO_2_^−^ to the toxic gases nitrous acid (HNO_2_) and NO via chemodenitrification (22).

Here, we delved into the nitrogen metabolism of “*Ca*. Na. tergens” to unravel the mechanisms of ammonia oxidation at both acidic and neutral pH. To investigate its physiology, we studied the activity and N-dynamics across a pH range from 2.5 to 7.5. Additionally, we examined its transcriptomic response to identify changes in the ammonia oxidation pathway and its response to acid stress. This research combined extensive chemostat cultivations of a highly enriched “*Ca*. Na. tergens” culture with metagenomic, metatranscriptomics, and nitrogen stable isotope analyses, enabling detailed monitoring of both biological and chemical N-conversion pathways of acidic ammonia oxidation.

## MATERIALS AND METHODS

### Cultivation

The biomass used in this study originated from a stable, highly enriched chemostat culture of “*Ca*. Na. tergens” sp. RJ19, which was isolated from a biological air scrubber, as described earlier (6). The organism’s genome is publicly available (NCBI genome assembly GCA_902810445.1). The culture medium consisted of 70 mM NH_4_Cl, 1 mM NaCl, 0.8 MgSO_4_·7H_2_O, 1 mM K_2_HPO_4_, and 0.2 mM CaCl_2_·H_2_O. After autoclavation, chelated trace elements were added to the medium with a final concentration of 18 µM FeSO_4_·7 H_2_O, 1.5 µM ZnSO_4_·7H_2_O, 1 µM CoCl_2_·6H_2_O, 5 µM MnCl_2_·4H_2_O, 1 µM CuSO_4_·5H_2_O, 0.9 µM Na_2_MoO_4_·2H_2_O, 1.5 µM NiCl_2_·6H_2_O, 0.6 µM NaSeO_4_, 2.3 µM H_2_BO_3_, and 0.6 µM CeCl_3_·7H_2_O. The bioreactor was operated as described in detail previously (6). In short, it was controlled via an ADI 1010 biocontroller (Applikon Biotechnology B.V., Delft, The Netherlands) and equipped with pH, dissolved oxygen (DO), and level sensors, maintaining a liquid level of 2.2 L. The pH of the culture was automatically maintained at pH 4.0 by dosing sterile 1 M KHCO_3_. The reactor was operated at room temperature and continuously stirred at 850 rpm. The medium was supplied at a rate of 1 L/day, and biomass from the bioreactor was removed at a rate of 200 mL/day. The remaining 800 mL spent medium was removed via a membrane filter.

A second bioreactor was inoculated with the biomass from the “parent” bioreactor and maintained at pH 4.0 until stable carbonate consumption was observed. As ammonia oxidation acidifies the medium, carbonate consumption was used as a proxy of activity. This was recorded automatically by placing a bottle containing 1 M KHCO_3_ on a balance connected to a computer, with weight measurements logged every 5 min using SPDC Data Collection software (Ohaus Corporation, New Jersey, USA). Once the culture reached a steady state, the pH of the bioreactor was lowered from 4.0 to 3.0 in steps of 0.1 during the period of a month, while carefully monitoring carbonate consumption of the culture. Finally, after the bioreactor ran steadily for a month on pH 3.0, we brought the pH to 2.5 4 days before the start of the experiment. During the experiment, 5 mL of biomass was daily sampled for nitrogenous compounds analysis (NH_4_^+^, NO_2_^−^, and NO_3_^−^) and protein determination. Biomass was vortexed, ensuring an equal distribution of biomass, and three 1 mL aliquots were centrifuged at 20,000 × *g* for 5 min. The supernatant from two of the replicates was directly diluted 1:1 with phosphate buffer (400 mM KH_2_PO_4_/400 mM K_2_HPO_4_; pH 7.4) to prevent the chemical conversion of NO_2_^−^. The biomass pellets, undiluted supernatant, and the diluted, buffered supernatant were stored at –20°C until analysis. In addition to these liquid samples, the N_2_O concentration in the off-gas of the bioreactor was also determined daily. Analytical assays and gas measurements are described in the sections below.

**Fig 1:**
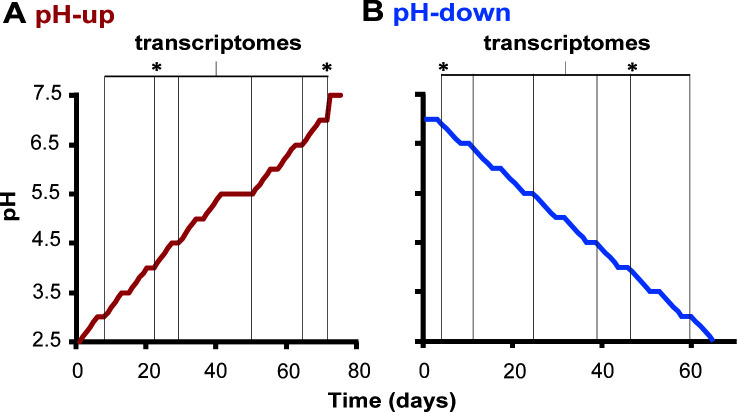
Schematic representation of the experimental setup of the “*Candidatus* Nitrosacidococcus tergens” enrichment culture bioreactor. The bioreactor was restarted at pH 4.0 between the pH-up (**A**) and pH-down (**B**) runs and was brought to pH 2.5 (**A**) or 7.0 (**B**). Black lines indicate the time/pH points for transcriptome sequencing (pH 3.0, 4.0, 4.5, 5.5, 6.5, and 7.0); asterisks indicate samplings for metagenomic sequencing (pH 4.0 and 7.0).

After daily sampling, the pH set point of the bioreactor was increased by 0.1 every weekday until pH 7.5 was reached. During weekends, no pH changes were made, and the biomass was harvested for metagenome and metatranscriptome analyses every Monday morning (Fig. 1A) in 15 mL tubes; the biomass was immediately centrifuged (5 min, 5,000 × *g*, RT). The pellets were snap-frozen with liquid nitrogen and stored at −70°C until further processing. During the pH-up run at pH 5.5, the medium inflow to the bioreactor was accidentally blocked for 24 h, which caused a severe drop in activity. Therefore, we ran the bioreactor at pH 5.5 for 1 week, until activity equaled pre-interruption levels, before the experiment was resumed (Fig. 1A).

At the end of the pH-up run, the bioreactor was restarted with fresh biomass from the “parent” bioreactor. When the bioreactor was running steadily at pH 4.0 with constant carbonate consumption, the pH was brought up to pH 7.0 in steps of 0.5, in a period of 2 weeks. The experiment was then conducted in reverse, decreasing the pH from 7.0 to 2.5 (pH-down run; Fig. 1B).

### Ammonium determination

Total ammonium of the bioreactor samples was determined colorimetrically at 420 nm after 20 min reaction of 10 µL sample (diluted to 0.5–5 mM NH_4_^+^) with 150 µL of 0.54% (wt/vol) ortho-phthalaldehyde with 0.05% (vol/vol) β-mercaptoethanol and 10% (vol/vol) ethanol in 400 mM, pH 7.4 potassium phosphate buffer (23).

### Nitrite and nitrate determination

Nitrite and nitrate concentrations were determined from the diluted phosphate-buffered supernatant samples by anion exchange chromatography on a Dionex ICS-2100 (Dionex, USA). The chromatograph was equipped with a Dionex IonPac AS19 column (Dionex, USA) operated at 30°C and a suppressed conductivity detector. As eluent, a KOH concentration gradient from 10 to 40 mM was used at a flow rate of 0.4 mL min^−1^.

### NO determination

NO concentration was measured in the bioreactor’s off-gas using a Nitric Oxide Analyzer (Sievers NOA 280i; Zysense, Frederick, CO, USA). The off-gas was connected via PVDF tubing to the inlet of the NOA 280i and measured every minute. The machine was calibrated using pure NO gas diluted with N_2_ gas or air using mass flow controllers. The average NO concentration was calculated per hour.

### N_2_O determination

N_2_O concentrations in the bioreactor off-gas were measured daily by sampling 100 µL gas with a gas-tight syringe (Hamilton, Reno, NV, USA) via a septum-sealed glass vial integrated into the off-gas line. N_2_O in the gas sample was measured with a 6890A gas chromatograph equipped with an Electron Capture Detector (Agilent Technologies, Santa Clara, CA, USA).

### Protein extraction and determination

Biomass (1 mL of bioreactor culture in triplicate) was pelleted (5 min, 20,000 × *g*), and the supernatant was removed. The pellet was resuspended in 50 µL of 1× PBS pH 7.4 (130 mM NaCl, 10 mM phosphate buffer), after which 50 µL of 2 M NaOH was added to lyse the cells and extract the proteins. The samples were incubated for 5 min at 90°C, and 100 µL 1 M HCl was added once the samples cooled down to neutralize the suspension, resulting in a 5-fold concentrated protein solution. The cell lysate was centrifuged (1 min, 20,000 × *g*) to pellet the remaining cell debris, and the dissolved proteins were transferred to a fresh tube. Protein concentration was determined using the copper-based BCA Protein Assay (Thermo Fisher Scientific, Waltham, MA, USA).

### Nucleic acid extractions and sequencing

Biomass for DNA and RNA extractions was harvested from the bioreactor in 15 mL tubes by centrifugation (5 min, 5,000 × *g*, RT). Pellets were snap-frozen with liquid nitrogen and stored at −70°C until further processing.

Total DNA was extracted, and metagenomes were sequenced for both pH-up and pH-down runs at pH 4 and 7. DNA extractions were performed in duplicate using the DNeasy Blood & Tissue Kit (Qiagen, Valencia, CA, USA), following the manufacturer’s instructions. DNA quantity was determined with a Qubit 2.0 fluorometer (Thermo Fisher Scientific) using the dsDNA HS Assay Kit. After quantitation, duplicate samples were pooled equally to ensure sufficient DNA quantity. Metagenomic libraries were prepared using the TruSeq DNA PCR Free Kit (350 bp insert) and pair-end sequenced (2 × 150 bp read length) on a NovaSeq 6000 platform (Illumina, San Diego, CA, USA) by Macrogen Inc. (Amsterdam, the Netherlands).

Total RNA was extracted, and transcriptomes were sequenced for both pH-up and pH-down runs at pH 3, 4, 4.5, 5.5, 6.5, and 7.0. Total RNA was extracted and purified in three technical replicates using the TRIzol Plus RNA Purification Kit and Phasemaker Tubes Complete System (Thermo Fisher Scientific). Manufacturer’s guidelines were followed, with minor modifications: 700 µL TRIzol was added to the thawed biomass pellets, and an incubation step of 10 min at 60°C was added. Once the samples were cooled to room temperature again, the biomass suspended in TRIzol was transferred to the Phasemaker Tubes, and 140 µL chloroform was added. Total RNA was eluted in two steps from the spin cartridges using 15 µL RNase-free water each time. Remnants of DNA were removed using DNase I from the DNA-*free* DNA Removal Kit (Thermo Fisher Scientific) following the manufacturer’s guidelines. Quality and quantity of total RNA were assessed using a Bioanalyzer 2100 (Agilent, Santa Clara, CA, USA) and the Qubit using the RNA HS kit (Thermo Fisher Scientific).

Total RNA samples were depleted of ribosomal RNA with the NEBNext rRNA Depletion Kit suitable for bacterial samples, and cDNA libraries were prepared using the TruSeq Stranded Total RNA kit (Illumina, San Diego, CA, USA) and were pair-end sequenced (2 × 100 bp) on an Illumina NovaSeq 6000 system (Macrogen Inc., Amsterdam, Netherlands).

### Metagenome and metatranscriptome analyses

Metagenomic read quality was assessed with FastQC v0.11.9 (24) before and after trimming. Using BBTools v38.75 (25), sequencing adapters were removed, and the reads were quality-trimmed with BBDuk, followed by error correction using Tadpole v39.06 and read normalization using BBNorm. Subsequently, the four samples were co-assembled in metaSPAdes v3.15.5 (26). Sequencing coverage for contigs ≥ 1,000 bp was calculated by mapping the non-normalized reads from each sample separately to the assembly with BBMap v38.75 (27). These contigs were then binned into metagenome-assembled genomes (MAGs) using four binning tools: CONCOCT v1.1.0 (28), MaxBin2 v.2.2.7 (29), MetaBAT2 v.2.12.1 (30), and BinSanity v0.4.4 (31), followed by consensus binning with DAS Tool v1.1.1 (32) and taxonomic classification by GTDB-Tk v2.1.1 (33). Genomic coverage of the MAGs was calculated using the CheckM v.1.1.2 coverage and profile functions (34). MAG completeness and contamination were estimated with CheckM2 v.1.0.1 (35). Genome annotation was done with Metascan v.1.2 (36) and METABOLIC v4.0 (37). A functional analysis of protein signatures was performed using InterPro (38).

Metatranscriptomic read quality was assessed with FastQC v0.11.9 (24), and the reads were quality-trimmed using Sickle v1.33 (39) with the paired-end option (pe) and the Sanger sequencing option (-t sanger). Trimmed RNA reads were mapped against the annotated coding DNA sequences (CDS) of the annotated MAGs using Kallisto v0.50.1 (40). Instead of mapping against the obtained “*Ca*. Na. tergens” MAG, we used the circularized RefSeq genome (NCBI Reference Sequence GCF_902810445.1). Transcripts per million (TPM) values were calculated from mapped read counts and gene length to compare gene expression profiles within and between samples. For analysis of the *“Ca*. Na. tergens” transcriptome only, transcripts mapping to *“Ca*. Na. tergens” were selected, and TPM values of the “*Ca*. Na. tergens” transcriptome were recalculated. Differential gene expression was assessed using the *voom* function of the limma R package (41, 42), which tests for statistical differences in gene expression patterns. Log_2_ fold changes (L_2_FC) ≥ 1 with an adjusted *P* < 0.01 were considered significantly differentially expressed. Hierarchical clustering and a principal component analysis were performed on the normalized gene expression profiles of “*Ca*. Na. tergens” using hclust and prcomp function of the stats package. All scripts used for the metatranscriptome analysis are made available via the Zenodo repository (https://doi.org/10.5281/zenodo.18850039).

### Microscopy

Biomass composition and growth state were monitored weekly as wet mounts with light microscopy using a Zeiss Axioplan 2 microscope (Carl Zeiss AG, Oberkochen, Germany). Pictures were taken with an AxioCam (Zeiss).

### Nitrogen stable isotope analysis

During the pH-down run (pH 6.0 to 3.0 in 0.5-unit increments), 20 mL of biomass was harvested, divided over 1.5 mL microcentrifuge tubes, and centrifuged for 5 min at 20,000 × *g*. The supernatant was filtered using a 0.2-µm Whatman GD/X syringe filter and used for stable isotope analysis as described in the Supplemental Materials and Methods.

## RESULTS

### Stoichiometric NH_4_^+^ conversion to NO_2_^−^ above pH 6.0

**Fig 2:**
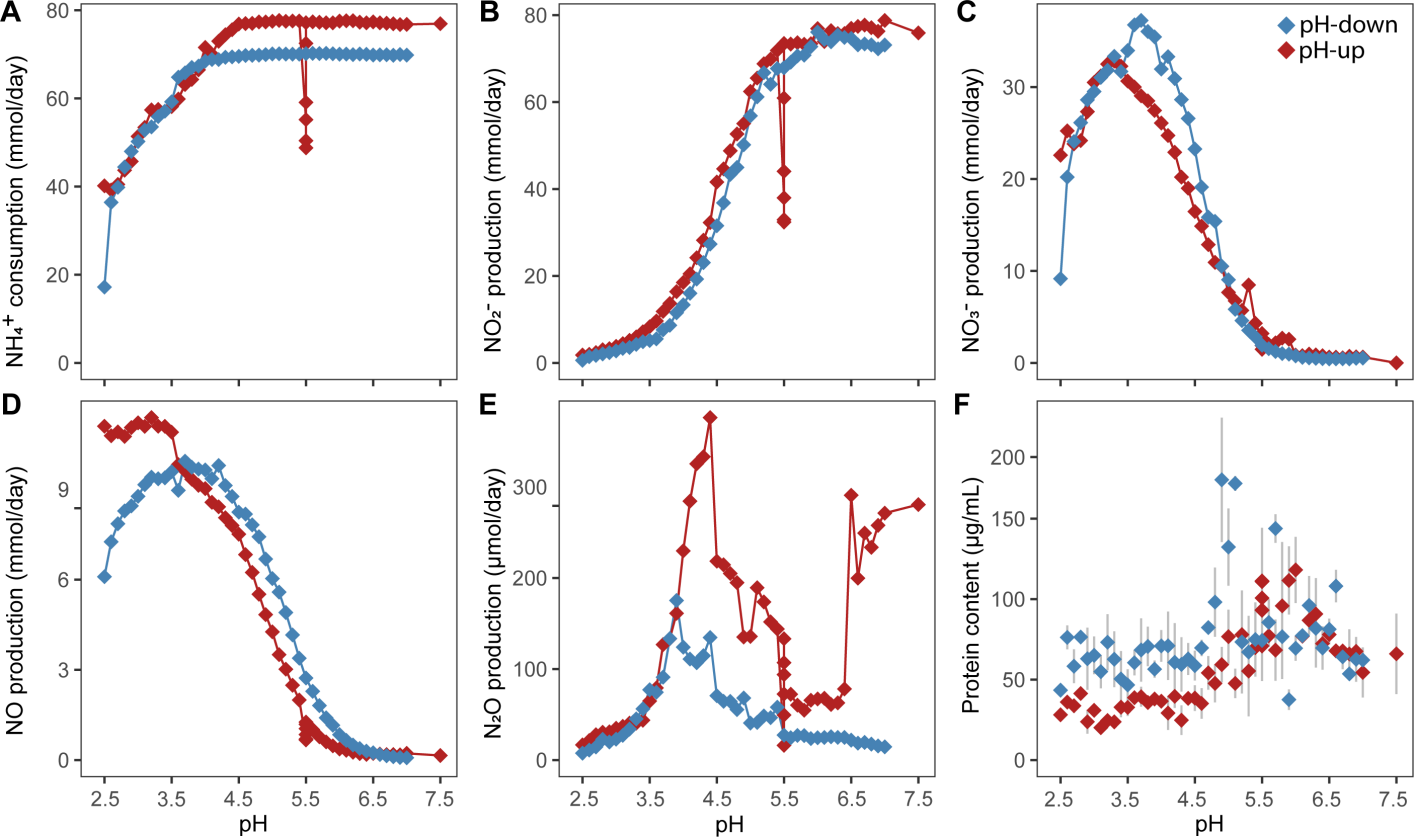
Daily measurements of the nitrogen dynamics of “*Ca*. Na. tergens” sp. RJ19 enrichment bioreactor cultures during the pH-up (red) and pH-down (blue) bioreactor runs. (**A**) NH_4_^+^ consumption and production of (**B**) NO_2_^−^, (**C**) NO_3_^−^, (**D**) NO, and (**E**) N_2_O. Rates are displayed in mmol/day except for N_2_O production, which is shown in µmol/day. (**F**) Protein content of the biomass in the bioreactor, determined in triplicate and shown in µg/mL; standard deviation is indicated with gray bars. See Table S1 for numerical data corresponding to these graphs. Note: Due to medium inflow failure at pH 5.5 (pH-up), NH_4_^+^ oxidation activity was reduced in the bioreactor, and the experiment was paused. The experiment was continued after 1 week of recovery.

During two experimental bioreactor runs with the enrichment culture *“Ca*. Na. tergens,” the pH was changed stepwise, first increasing from 2.5 to 7.5 (Fig. 1A) and decreasing from 7.0 to 2.5 during the second run (Fig. 1B). Throughout the experiments, the culture continuously received 70 mM NH_4_^+^. NH_4_^+^ was consumed completely above pH 5.0 but partially accumulated under more acidic conditions (Fig. 2A and B; Fig. S1; Table S1). Above pH 6.0, NH_4_^+^ was stoichiometrically converted to NO_2_^−^, whereas below this pH, NO gas and NO_3_^−^ were formed (Fig. 2C and D). During the pH-up run, between pH 2.5 and 3.5, NO concentration was the highest (13.7%–14.5% of total N), while at pH 3.3, NO_3_^−^ peaked (42% of total N; Fig. S1a). During the pH-down run, NO and NO_3_^−^ maxima (14.1% and 52.8% of total N, respectively) shifted slightly toward higher pH (both at pH 3.7) compared to pH-up, and NO concentration declined strongly below pH 3.7 (Fig. 2C and D; Fig. S1b).

The decline in NO production and the detected NH_4_^+^ accumulation toward low pH indicated less NH_4_^+^ oxidation activity. Indeed, the ammonia-oxidizing activity, followed as KHCO_3_ consumption, slowly declined under acidic conditions and finally dropped drastically during the pH-down run at pH 2.5 (Fig. S2), with only minor NH_4_^+^ conversion into other N-compounds (Fig. 2A; Fig. S1). During the pH-up run, protein content started to increase above pH 4.5 and peaked at pH 6.0. In the pH-down experiment, overall protein content up to pH 5.0 was slightly higher than that during the pH-up run and peaked around pH 5.0 (Fig. 2F).

Furthermore, N_2_O concentration in the off-gas of the bioreactors was low (10–375 µmol/day) compared to other N compounds. Concentrations peaked between pH 3.5 and 4.5, with 2-fold lower amounts in the pH-down run (Fig. 2E). In addition, the pH-up run showed a second N_2_O production peak between pH 6.5 and 7.5. Finally, the N-balances were incomplete below pH 5.0 (Fig. S1), likely because NO_2_/HNO_2_ concentrations were not determined during this experiment. The presence of these gases in the off-gas of the “parent” bioreactor at pH 4.0 was confirmed (43).

### Metagenomic and metatranscriptomic analyses show dominance of “*Ca.* Na. tergens”

**Fig 3:**
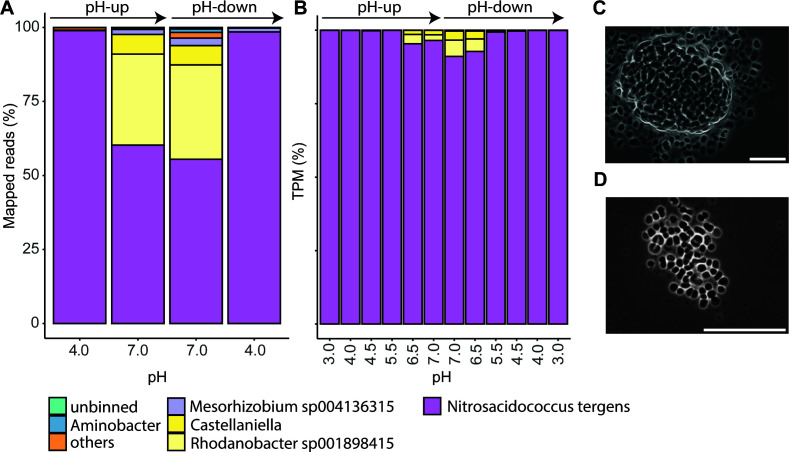
Community composition in the enrichment bioreactor. Metagenomic (**A**) and metatranscriptomic (**B**) analyses of the microbial community in the “*Ca*. Na. tergens” enrichment culture during the pH-up and pH-down experiments (indicated with arrows on top of the bar chart). Metagenomic read percentages are based on read-mapping percentages to each binned MAG, and metatranscriptomic percentages refer to normalized transcripts per million (TPM). Species with <1% abundance are grouped in “others.” Light microscopic pictures of the enrichment culture at pH 4.1 (**C**) and 7.0 (**D**), with scale bars indicating 50 µm.

During the pH-up and pH-down bioreactor runs, “*Ca*. Na. tergens” was the dominant species and the only ammonia oxidizer based on MAG classification (Table S2), a screening of the metabolic potential of the side population (Table S3), and quantifications of metagenomic and transcriptomic reads (Fig. 3A and B). “*Ca*. Na. tergens”-associated metagenomic reads at pH 4.0 constituted 98.9% during the pH-up and 98.5% during the pH-down experiment. In contrast, at pH 7.0, only 60.3% and 55.5%, respectively, of the sequenced reads mapped to the acidic AOB genome, indicating that contaminating species were able to rapidly proliferate in neutral pH conditions (Fig. 3A and B). Contrastingly, >91% of metatranscriptomic reads mapped to “*Ca*. Na. tergens” in all conditions, with even 99% for the pH 3.0–5.5 conditions. Therefore, we focused on the gene expression patterns of “*Ca*. Na. tergens” in this study (Table S4; see Table S5 for TPM values of the whole community).

Additionally, changes in biomass composition were inspected visually by light microscopy. Large cell aggregates of the large (diameter ± 500 nm) and characteristically shaped “*Ca*. Na. tergens” were visible at acidic pH conditions (Fig. 3C). This shifted to smaller aggregates and single cells of “*Ca*. Na. tergens” at neutral pH, and smaller rod-shaped side populations became visible (black dots in Fig. 3D). Additionally, during acidic pH conditions, the cells of “*Ca*. Na. tergens” were larger in volume compared to neutral pH (Fig. 3C and D).

### Genomic potential of the most abundant side population members

At pH 7, the most dominant side population of *“Ca*. Na. tergens” was composed primarily of *Mesorhizobium* sp. (1.6%–2.6%), *Castellaniella* sp. (6.5%–6.7%), and *Rhodanobacter* sp. (30.7%–31.8%) (Fig. 3A), none of which encodes CO_2_ fixation pathways (Table S3), indicating that they all are heterotrophic organisms proliferating on metabolic exudates, extracellular polymeric substances, or dying biomass from *“Ca*. Na. tergens.” While no dissimilatory nitrogen cycling potential was detected in the *Mesorhizobium* sp. bin, we identified nitrate reductase genes *narGHI* (CNOMCMLN_01321/01322/01324) in the *Castellaniella* bin. Additionally, it has two copies of a NO-producing nitrite reductase (*nirK;* CNOMCMLN_00162/03060, 30% protein identity) and the nitric oxide reductase NorB, which produces N_2_O (*norBC;* CNOMCMLN_02442/02760). Finally, the *Rhodanobacter* MAG also contained denitrification genes, including two copies of *nirK* (LNGJGBKO_01687/02535; 37% shared protein identity) and *norBC* (LNGJGBKO_02472/02506). A comprehensive overview of the metabolic potential of the side population is provided in Table S3.

### Transcriptomic changes in the nitrogen metabolism of *“Ca*. Na. tergens”

Overall, the gene expression profile of “*Ca*. Na. tergens” was slightly influenced by pH changes (Table S6). Of all 1,706 annotated coding genes, 141 and 83 were upregulated, and 103 and 112 were downregulated during the pH-up and pH-down runs, respectively, when comparing pH 3.0 to pH 7.0, which were the two conditions with the highest number of differentially expressed genes. Expression profiles of pH conditions 3.0, 4.0, and 4.5 cluster together for the pH-up and pH-down runs (Fig. S3), indicating similar gene expression patterns across the two pH gradients. Contrastingly, profiles at pH 5.5, 6.5, and 7.0 were more distinct between experiments (Fig. S3a), with the pH-up profiles being most divergent (Fig. S3b).

**Fig 4:**
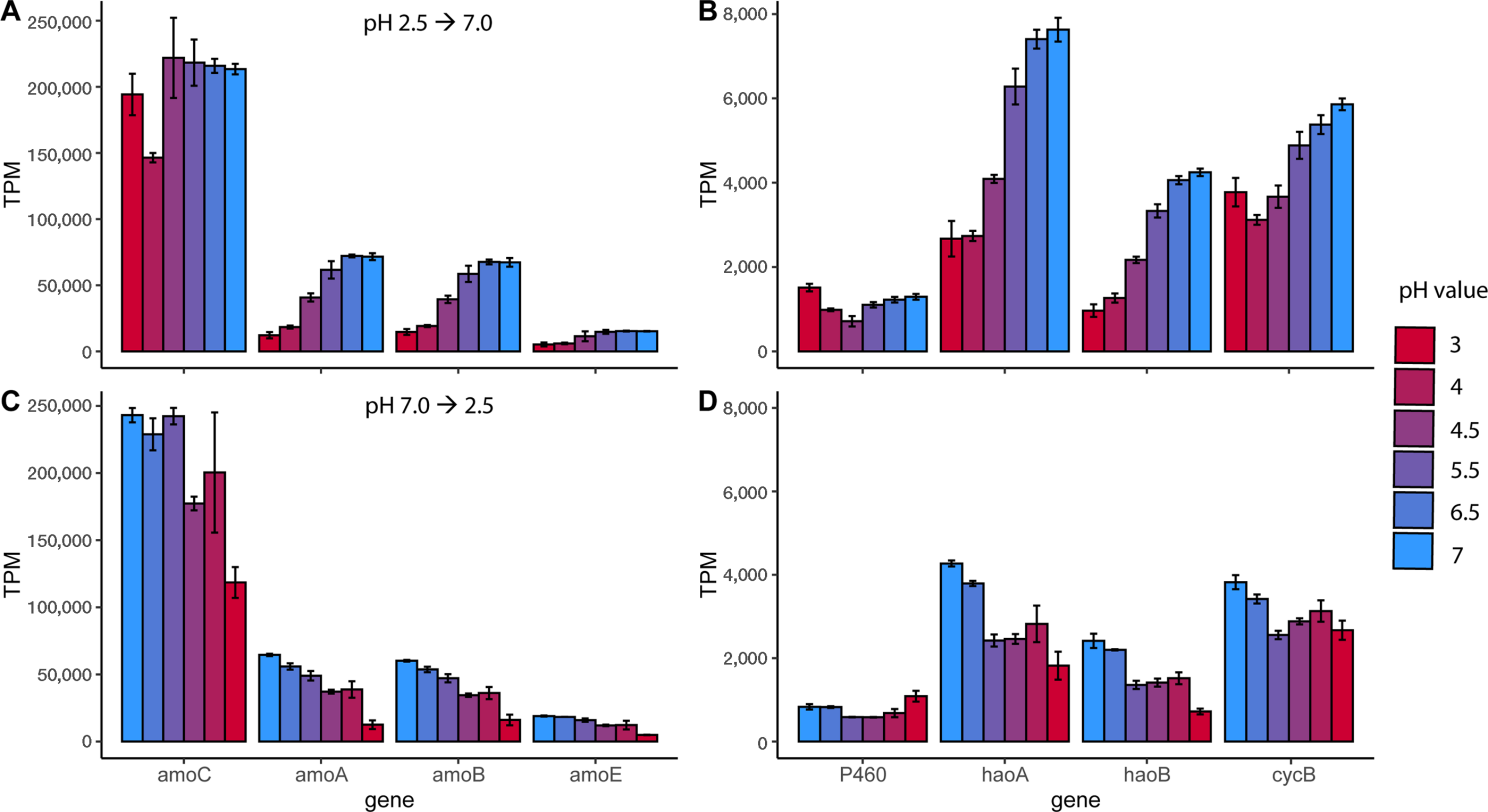
Transcripts per million (TPM) of genes in the ammonia monooxygenase operon (**A and C**) and hydroxylamine oxidoreductase gene cluster (**B and D**) during the pH-up (**A and B**) and pH-down (**C and D**) experiments. Bars represent the average of technical-replicate transcriptomes (*n* = 3), with pH indicated by a red (pH 3) to blue (pH 7) color gradient; error bars indicate the standard deviation. Statistical significance of pairwise comparisons between conditions is reported in Table S4.

In both pH-up and pH-down experiments, the highly transcribed ammonia monooxygenase (*amoCABD*) operon (NSCAC_1280-NSCAC_1276) was significantly upregulated at pH 7.0 compared to pH 3.0 (Fig. 4; Table S4; L_2_FC > 2.0, *P* < 0.001), except for *amoC* during the pH-up run. The significant change during the pH-up experiment occurred for *amoAB* between pH 4 and 4.5 (L_2_FC = 1, *P* < 0.001), whereas for the pH-down run, this occurred for amo*ABD* between pH 4 and 3 (L_2_FC > 1.4, *P <* 0.001). No significant change in the expression levels was observed for the other pH conditions. Following the same trend as the AMO operon, *cycB* (NSCAC_1125) and *haoAB* (NSCAC_1126 and 1127) were upregulated at pH 7.0 compared to 3.0 (Fig. 4; L_2_FC = 1.0-2.6, *P* < 0.001). Finally, no differential gene expression was detected for the cytochrome P460 (NSCAC_1129), which is in the same gene cluster as the *haoAB-cycB*.

**Fig 5:**
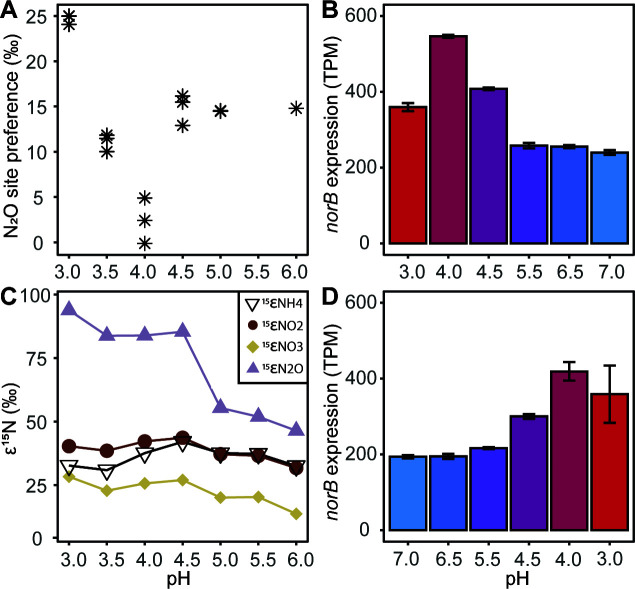
Site preference of the N_2_O molecule (**A**) defined as SP = δ^15^Nα – δ^15^Nβ and natural isotope effects of nitrogen species (**C**) during the pH-down experiment at pH 6.0, 5.5, 5.0, 4.5, 4.0, 3.5, and 3.0; values are expressed in per mille (‰) with ^15^ε_NH4_ referring to NH_4_^+^reactor – NH_4_^+^inflow, ^15^ε_NO2_ referring to NH_4_^+^ – NO_2_^−^, ^15^ε_NO3_ referring to NH_4_^+^ – NO_3_^−^, and ^15^ε_N2O_ referring to NH_4_^+^ – N_2_O. Expression profiles of the *norB* gene in transcripts per million (TPM) of *“Ca.* Na. tergens” during the pH-up (**B**) and pH-down runs (**D**). Bars represent the average of triplicate transcriptomes, with pH indicated by a red (pH 3) to blue (pH 7) color gradient; error bars indicate standard deviation.

Nitrosocyanin (NSCAC_0629), previously proposed as a possible nitric oxide dehydrogenase (13, 16), was highly expressed in all pH conditions and follows similar expression patterns to other ammonia oxidation genes, suggesting a role in NH_3_ oxidation (Fig. S4A and B). In contrast, the *“Ca.* Na. tergens” genome encodes a nitric oxide oxidoreductase (*norB*; NSCAC_1563), and unlike the nitrification genes, the expression of this denitrification gene peaked at pH 4.0 and was the lowest at pH 7.0 in the pH-up and pH-down runs (Fig. 5). Finally, a gene previously annotated as ammonia permease (NSCAC_1037) is among the highest expressed genes (Fig. S4C and D), especially during the pH-down run. However, the predicted structure of this small protein (www.uniprot.org/uniprotkb/A0A7G1QAP4) shows no accessible transmembrane channel, making NH_3_/NH_4_^+^ transport unlikely. Furthermore, no functional domains were identified in this sequence. However, as the transcriptional levels are almost as high as the AMO operon, it appears to have an important function in the metabolism of “*Ca*. Na. tergens.”

### Site preference indicates nitrifier-denitrification as an N_2_O source at pH 4.0

To better understand the N transformations occurring during acidic ammonia oxidation, we measured N isotopic signatures and calculated the corresponding isotope effects across the pH-down experiment (Fig. 5; Fig. S5). The δ^15^N of the supplied NH_4_^+^ was −0.67‰, while the NH_4_^+^ remaining in the reactor averaged at 31‰ ± 5‰, resulting in a ^15^ε_NH4+_ of 28‰ at both low and high pH. At pH 4–5.5, ^15^ε_NH4+_ increased to 33‰–38‰ (Fig. 5C; Fig. S5). The isotope effect associated with NO_2_^−^ production (^15^ε_NO2−_) closely matched the ^15^ε_NH4+_ above pH 5.0, ranging between 27‰ and 33‰. However, at pH 4.5, these values began to diverge, with the ^15^ε_NO2−_ rising to 34‰–40‰, coinciding with the observed deviation from stoichiometric conversion of NH_4_^+^ to NO_2_^−^ and increased residual NH_4_^+^ in the reactor (Fig. 2A). Despite this divergence, both estimates of the isotope effects, ^15^ε_NH4+_ and ^15^ε_NO2−_, remained within the range reported for ammonia oxidation (44 and references therein). The isotope effect for NO_3_^−^ production (^15^ε_NO3^−^_) increased steadily from 7‰ at pH 6 to 23‰ at pH 3 and was consistently lower than the estimated isotope effects for NH_4_^+^ consumption and NO_2_^−^ production. To our knowledge, this is the first report of isotope effects for presumably abiotic NO_3_^−^ production during ammonia oxidation at acidic conditions. The bulk N isotope effect for N_2_O production (^15^ε_N2O_) also increased with decreasing pH, rising from 43‰ to 96‰, with a pronounced jump between pH 5 and 4.5 (Fig. 5C), which likely points to changing substrates (i.e*.*, N_2_O is formed from 2 NO, or 1 NO and 1 NH_2_OH below pH 4.5, instead of from 2 NH_2_OH above pH 5), as well as a shift in production mechanisms.

The N_2_O site preference (SP) varied between 2.4‰ and 25‰ across the tested pH range, which is lower than the >30‰ found for canonical nitrification (45–48). The SP at pH 3.5 and between pH 4.5 and 6.0 ranged between 11.1‰ and 14.8‰, likely corresponding to abiotic reactions between NO_2_^−^ or NO with Fe(II), as such reactions display a wide SP range between 0‰ and 30‰ (49–53). SP values for N_2_O produced by denitrification, including nitrifier denitrification, range mostly between −11‰ and 0‰ (45–47, 54). Interestingly, a distinct minimum at pH 4.0 (2.4‰ ± 2.5‰) corresponded with elevated expression of *norB* (Fig. 5B and D), indicating nitrifier-denitrification by “*Ca.* Na. tergens” as the dominating source of the N_2_O. Expression of *norB* by members of the side population can be ruled out (Table S7), as the only *norB* transcripts detected below pH 5.5 originated from the acidic AOB. Finally, a distinct maximum SP of 24.7‰ ± 0.5‰ was measured for pH 3.0, falling within the SP range corresponding to abiotic reactions.

### Acid stress adaptation of “*Ca*. Na. tergens”

The general stress response sigma factor RpoS (NSCAC_0889) was strongly upregulated at pH 3.0 compared to pH 7.0 in both pH-up and pH-down runs (L_2_FC of 3 and 5, *P* < 0.001), indicating activation of a broad stress response. In contrast, canonical pH homeostasis genes, including K^+^ transporters (*trkA,* NSCAC_0059; *trkH*, NSCAC_1602), Na^+^/H^+^ antiporters (*nhaA,* NSCAC_0872), and other predicted antiporters (NSCAC_1736 and NSCAC_1738), were expressed at low levels and not differentially expressed across the pH range (for an extensive overview of acid stress response mechanisms, see Table S8). Chaperonin-encoding genes, which are involved in protein folding or stabilization (*groES* and *groEL*, NSCAC_0031–NSCAC_0032), were slightly downregulated at pH 3.0 compared to pH 7.0 (L_2_FC = 1–2, *P* < 0.001), whereas the expression of HSP20-like chaperone peaked at pH 4.0. The catalytic subunit of urease (*ureC*; NSCAC_0479), a nickel-dependent metalloenzyme producing NH_3_ and bicarbonate (HCO_3_^-^) for potential cytoplasmic buffering, was slightly upregulated at pH 3.0 compared to pH 7.0 (L_2_FC = 1, *P* < 0.001) in both runs. The other urease subunits, *ureAB* (NSCAC_0477–0478), showed similar regulation but were overall more highly expressed. The accessory genes *ureDFG* (NSCAC_0476, NSCAC_0481, NSCAC_0482) followed a similar trend, although *ureF* had low transcript levels (max. TPM = 40). Additionally, the ferric uptake regulator *fur* (NSCAC_1189) was significantly upregulated at low pH (L_2_FC = 1.0, *P* < 0.001). In contrast, gene expression of the TonB systems, involved in iron transport over the outer membrane and typically repressed by *fur*, was upregulated at neutral pH (Table S8). Finally, genes involved in canonical acid stress pathways, including fatty acid synthesis, branched chain amino acid synthesis (fatty acid precursor), DNA repair, cell division, and extracellular polymeric substance production, showed overall low expression and no significant differences across pH conditions. This lack of a pronounced differential expression, despite a 10,000-fold increase in proton concentration between pH 7.0 and 3.0, suggests the “*Ca.* Na. tergens” has an exceptional acid tolerance, likely relying on constitutive expression combined with post-transcriptional regulation and adjustment in membrane permeability.

## DISCUSSION

To gain deeper insights into the microbial ammonia oxidation pathway under acidic conditions, we conducted a bioreactor experiment where pH was first gradually increased from 2.5 to 7.5 and subsequently decreased from pH 7.0 to 3.0 while monitoring chemical and transcriptomic changes of the acidophilic ammonia oxidizer “*Ca.* Nitrosacidococcus tergens” sp. RJ19. This approach allowed us to explore nitrogen conversions catalyzed by “*Ca*. Na. tergens” across a broad pH range, particularly in neutral conditions where its ammonia-oxidation activity had not yet been extensively studied. Understanding the physiology and pH adaptability of acid-tolerant AOB like “*Ca*. Na. tergens” is essential for optimizing nitrification processes in engineered systems like wastewater treatment (9).

### Nitrogen metabolism and pH effects

Our findings confirm that “*Ca*. Na. tergens” performs canonical ammonia oxidation at neutral pH, despite lacking the *cycA* and *nirK* genes that are omnipresent in AOB genomes (20). Their absence in multiple gammaproteobacterial AOB genomes (5, 8) confirms that these genes are not essential for ammonia oxidation and thus are also not involved in NO oxidation, a process for which the mechanism in ammonia oxidizers remains unresolved (19). The absence of *cycA*, which encodes the cytochrome *c*554 responsible for electron shuttling from the HAO complex to the quinone pool*,* in the “*Ca*. Na. tergens” genome, its associated community, and in the unbinned fraction raises the question of how acidophilic gammaproteobacterial NH_3_ oxidizers accomplish electron transport. However, Simon and Klotz (55) previously mentioned that since experimental evidence is lacking, a direct interaction between HaoA and cytochrome *c*_M_552 cannot be excluded. NirK can oxidize NO to NO_2_^−^ (18) and is suggested to be a possible candidate for the oxidation of NO to NO_2_^−^ (13, 19). However, a Δ*nirK* knockout *Nitrosomonas europaea* ATCC showed a normal doubling time, suggesting that NirK is not essential for nitrifier growth; nevertheless, the culture showed impeded NO_2_^−^ production, thereby implying a role in ammonia oxidation (56). In this study, we see stoichiometric conversion of NH_4_^+^ to NO_2_^−^ at neutral pH conditions. This indicates that *“Ca.* Na. tergens” and possibly also other (acidic) AOBs lacking NirK might have an adapted ammonia oxidation pathway independent of this enzyme.

The presence of NO and NO_3_^−^ in the bioreactor was mostly detected below pH 6.0. As the concentrations of these two compounds were minimal above pH 6.0, and their known chemical formation pathways with NO_2_^−^ as substrate under aerobic conditions indeed happen mainly at acidic pH (22), they likely are formed abiotically as a result of the protonation of NO_2_^−^ to HNO_2_. Indeed, it was determined in a previous study that the off-gas of our bioreactor running at pH 4.0 contained large quantities of NO_2_ and HNO_2_ (270 ± 40 ppm and 357 ± 2 ppm, respectively) compared to NO and N_2_O (89 ± 9 ppm and 11.2 ± 0.10 ppm, respectively) (43). Additionally, the formation of NO_2_ and HNO_2_ likely caused the gap in the calculated nitrogen balance, as these compounds were not measured during the experimental runs.

A peak in N_2_O production was detected between pH 3.5 and 4.5, and the upregulation of *norB* of “*Ca.* Na. tergens” at pH 4.0 correlates with the observed peak. Correspondingly, the SP of N_2_O produced at pH 4.0 is distinctly different from those at other pH values. It has a value between 0‰ and 5‰, which is indicative of denitrification (57). Therefore, we expect that chemically formed NO, which peaks around pH 4.0, is actively reduced by “*Ca*. Na. tergens” to N_2_O, under aerobic conditions. Notably, no *norB* of the side population was expressed under acidic conditions. Additionally, the observed SP of 11.1‰–24.7‰ for pH 3.0, 3.5, 4.5, 5.0, and 6.0 indicated that chemodenitrification is the origin of N_2_O (49–51). However, ^15^ε_N2O_, ^15^ε_NH4+_, and ^15^ε_NO2_ at pH 4.5–6.0 did not deviate from patterns expected from canonical ammonia oxidation. Therefore, we anticipated a nitrification-derived SP contributing to N_2_O production at pH 4.5–6.0, which was not observed. Possibly, N_2_O was produced through multiple co-occurring pathways, with denitrification (via NorB activity) and chemodenitrification being more dominant than nitrification and thereby dominating the observed lower SP values.

Notably, an elevated N₂O concentration was detected above pH 6.5 in the pH-up but not the pH-down run. This inconsistency in N_2_O concentration under similar pH conditions suggests biological rather than chemical N_2_O production. In the pH-up bioreactor run, in which N_2_O was elevated, either “*Ca*. Na. tergens” or the side community produced N_2_O via NorB in response to the nitrite-reductase possessing side community converting NO_2_^−^ into NO (Table S7). In our experimental setup, differences in timelines render the replicate runs not fully comparable. In the pH-up run, the reactor pH was first slowly lowered to pH 2.5 and subsequently increased stepwise, allowing the side community nearly two months to proliferate. Contrastingly, in the pH-down experiment, the adaptation from 4.0 to 7.0, as the starting point, was conducted within 2 weeks, thereby presumably limiting the adaptation of the side community. Because isotopic composition was only measured for the pH-down run, we cannot determine the origin of the N_2_O peak between pH 6.0 and 7.0 in the pH-up experiment.

### Stress response and acid adaptation

To maintain cellular function and bioenergetic stability under acidic conditions, “*Ca.* Na. tergens” likely preserves a stable cytoplasmic pH. As the proton motive force (PMF) depends on both the membrane potential (ΔΨ) and the transmembrane pH gradient (ΔpH), external acidification increases ΔpH and challenges the energetic balance (58, 59). In our study, most canonical stress-response and acid-response genes were expressed at low levels and showed no significant changes across the pH gradient. Indeed, membrane energetics and protein stability in extremophiles are often constitutively expressed (58), reflecting the pressure of extracellular pH as a persistent stressor. Moreover, acid stress responses are frequently partitioned into baseline maintenance and inducible, higher-cost mechanisms that respond to rapid pH shifts (60, 61).

In our study, upregulation of urease and accessory genes at low pH suggests a potential role of urease activity in pH homeostasis of “*Ca*. Na. tergens,” as it potentially counteracts acidification by hydrolyzing urea into NH₃ and bicarbonate (6). Although urea was not a substrate during our experiments, it is expected to be (temporally) present in environments where acidic AOB species like “*Ca.* Na. tergens” are isolated from (5, 6, 8, 62). Urease expression might be tightly regulated due to its energetic cost and its potential to disturb not only pH homeostasis but also the nitrogen and ion balance. Detected upregulation at low pH suggests that acidity itself acts as a primary regulatory signal, potentially in anticipation of neutralizable nitrogen compounds that are required for pH homeostasis, given the isolation environment of “*Ca*. Na. tergens” (6).

Additionally, organisms with small genomes, such as *“Ca.* Na. tergens*”* (1.8 Mbp), may rely on post-transcriptional regulation, membrane permeability adjustments, EPS production, or biofilm formation, explaining the absence of differentially expressed genes for these mechanisms (9, 58, 63). In line with this, both reduced nucleic acid extraction efficiency and observed morphology at low pH show the formation of large, less permeable aggregates impeding proton entry. Furthermore, the increased nitrous acid concentration at low pH imposes additional nitrosative stress alongside proton uncoupling, similarly to weak organic acids, which can disrupt membrane potential and bioenergetics (64). These findings highlight the need for integrative omics approaches to unravel the mechanisms underlying the acid tolerance of *“Ca.* Na. tergens*”* and AOB.

### Conclusion

This study highlights the exceptional robustness and acid tolerance of “*Ca*. Na. tergens” while maintaining ammonia oxidation across a wide pH spectrum. Over a pH range from 2.5 to 6.0, the pH strongly affected the nitrogen balances in the acid-adapted ammonia-oxidizing bioreactor. We found robust but less efficient NH_4_^+^ conversion under acidic conditions. Since “*Ca*. Na. tergens” is the dominant species at acidic pH and its genome does not encode a nitrite reductase, most NO and NO_3_^−^ were likely formed by abiotic processes. Isotopic signatures and gene expression data suggest that N_2_O is produced from a mix of NH_2_OH oxidation, NO reduction, and chemodenitrification, depending on pH and NO availability. To resolve the complex regulatory mechanisms underlying acid stress resilience in AOB, multi-omics could reveal mechanisms in post-transcriptional modification, membrane permeability, and EPS formation. Given the critical role of AOB in wastewater treatment, “*Ca*. Na. tergens” is a promising candidate for optimizing wastewater treatment at low pH, but only when the toxic gases NO and N_2_O can be captured or efficiently removed by concomitant microorganisms.

## Data Availability

All sequencing data produced in this study have been made available and can be found in the European Nucleotide Archive under the project accession number PRJEB95883. Additionally, high-quality MAGs and their corresponding protein annotations can be found under the same accession number. Lower quality bins and the unbinned fraction can be accessed via the Zenodo repository (https://doi.org/10.5281/zenodo.18850039).

## SUPPLEMENTAL MATERIAL

Supplemental information mbio.02975-25-s0001.docx Supplemental text, figures, and table captions; Table S6.

Supplemental tables part 1 mbio.02975-25-s0002.xlsx Tables S1 to S4.

Supplemental tables part 2 mbio.02975-25-s0003.xlsx Tables S5, S7, and S8.

ASM does not own the copyrights to Supplemental Material that may be linked to, or accessed through, an article. The authors have granted ASM a non-exclusive, world-wide license to publish the Supplemental Material files. Please contact the corresponding author directly for reuse.
