# Supplementary material for: Robust ammonia oxidation by “Candidatus Nitrosacidococcus tergens” across a broad pH range
Source: mBio. 2026 Apr 24;17(5):e02975-25. doi: 10.1128/mbio.02975-25 (PMC13170337; doi:10.1128/mbio.02975-25)
Supplement: Supplemental information — Supplemental text, figures, and table captions; Table S6. [file mbio.02975-25-s0001.docx]

**Supplementary information belonging to “Robust ammonia oxidation by “*Candidatus* Nitrosacidococcus tergens” across a broad pH range”**

Ida F. Peterse^1,2^, Claudia Frey^3^, Reinier A. Egas^1^, Guylaine H. L. Nuijten^1^, Annelies J. Veraart^2^, Sebastian Lücker^1#^

^1^ Department of Microbiology, Radboud Institute for Biological and Environmental Sciences, Faculty of Science, Radboud University, Nijmegen, the Netherlands

^2^ Department of Ecology, Radboud Institute for Biological and Environmental Sciences, Faculty of Science, Radboud University, Nijmegen, the Netherlands

^3^ Department of Environmental Science, University of Basel, Basel, Switzerland

^#^Correspondence: Sebastian Lücker ([s.luecker@science.ru.nl](mailto:s.luecker@science.ru.nl)) | Ida F. Peterse (i.peterse@science.ru.nl) |

The supplementary information contains the following content:

**Supplementary Material and Methods**

- Stable N isotope analysis

**Supplementary Figures**

- **Supplementary Figure 1** Nitrogen balances of “*Ca.* Na. tergens” sp. RJ19 enrichment bioreactor cultures fed with 70 mM NH_4_^+^
- **Supplementary Figure 2** KHCO_3_ consumption of the bioreactor culture
- **Supplementary Figure 3** Principal component analysis of normalized gene expression profiles of “*Ca*. Na. tergens and a dendrogram illustrating the hierarchical relationships between the samples
- **Supplementary Figure 4** Gene expression profiles of nitrosocyanin and hypothetical protein NSCAC_1037
- **Supplementary Figure 5** δ15N of N-compounds during pH-down run.

**Supplementary Tables**

- **Supplementary Table S1** Numeric values the nitrogen dynamics corresponding to the graphs in Figure 2.
- **Supplementary Table S2** Overview of metagenomic bins, stats, and GTDB-Tk classification (see separate Supplementary Tables excel file)
- **Supplementary Table S3** Overview of metabolic functions of each bin retrieved from METABOLIC (see separate Supplementary Tables excel file)
- **Supplementary Table S4** Gene expression in TPMs and differential gene expression of “*Ca.* Na. tergens” sp. RJ19 (see separate Supplementary Tables excel file)
- **Supplementary Table S5** Gene expression of the full “*Ca.* Na. tergens” sp. RJ19 enrichment culture (see separate Supplementary Tables excel file) in TPMs
- **Supplementary Table S6** Summary of differentially expressed genes per pairwise comparison within the pH-up and pH-down runs (this document)
- **Supplementary Table S7** Overview of *norB* and *nirK*/*nirS* expression of all community members (see separate Supplementary Tables excel file)
- **Supplementary Table S8** Gene expression and differential gene expression of “*Ca.* Na. tergens” genes involved in acid stress (see separate Supplementary Tables excel file)

Supplementary Material and Methods

During the pH-down run, 20 mL of biomass was harvested, divided over 1.5 mL microcentrifuge tubes, and centrifuged for 5 min at 20,000 × *g.* The supernatant was filtered using a 0.2 µm Whatman GD/X syringe filter after it was pre-rinsed with 5 mL demineralized water. The first 2 mL of filtered supernatant was discarded.

For NO_2_^-^ stable isotope analysis, NO_2_^-^ was reduced to N_2_O using azide/acetic acid reduction (1) as follows: Immediately after sampling, NO_2_^-^ concentrations were quantified using the Griess reaction (2) to accurately fix the right amount of NO_2_^-^ in the next step (40 nmol). Meanwhile, supernatant was subsampled in triplicate 5.9 mL exetainers (Labco, Lampeter, UK) and purged with N_2_ gas for 10 min to remove all N_2_O. Then, a volume of the purged supernatant corresponding to 40 nmol NO_2_^-^ (calculated above) was added to 3 mL of NO_2_^-^-free Low Nutrient Seawater (OSIL, UK) in a 20 mL serum vial. After capping the vial, NO_2_^-^ was fixed to N_2_O with 300 µL 2 M acetic azide (1:1 mixture of 2M NaN_3_ and 20% acetic acid). After vigorously shaking the vial, the reaction was stopped after ≥ 30 min by adding 300 µL 6M NaOH. Samples were stored at room temperature in the dark until further analysis.

For NO_3_^-^ isotope analyses, first all NO_2_^-^ was removed from the supernatant by adding 200 µL 0.6 sulfamic acid (H_3_NSO) to 1 mL of filtered supernatant to convert all NO_2_^-^ to N_2_ gas. The supernatant was neutralized after 5 min with 400 mM phosphate buffer pH 7.4 and stored at 4 °C until analysis. Supernatant for NH_4_^+^ analysis was directly frozen after the filtering. Additionally, 20 mL bioreactor off-gas was sampled for N_2_O isotope analysis by flushing a 120 mL serum bottle for 5 min after which it was capped with a red butyl stopper.

The N isotopic composition (δ^15^N) of NH_4_^+^ was determined by oxidizing NH_4_^+^ to NO_2_^-^ using hypobromite under alkaline conditions, followed by conversion of NO_2_^-^ to N_2_O via azide/acetic acid reduction as described above (3). For determining the δ^15^N of NO_3_^-^, all NO_3_^-^ was converted to N_2_O using the denitrifier method with *Pseudomonas chlororaphis* (subsp. *aureofaciens*) (4, 5). All N_2_O products were analyzed using GC-IRMS analysis (Delta V Plus, Thermo Fisher). Calibration standards included IAEA-N1 (δ^15^N = 0.40‰) and USGS26 (δ^15^N = 53.70‰) for NH_4_^+^, IAEA-N3 (δ^15^N = 4.70‰, δ^18^O = 25.61‰), USGS34 (δ^15^N = -1.59‰, δ^18^O = -27.93‰), and UBN-1 (δ^15^N = 14.15‰, δ^18^O = 24.19‰) for NO_3_^-^, and N-7373 (δ^15^N = -79.60‰) and N-10219 (δ^15^N = 2.80‰) for NO_2_^-^.

For N_2_O isotopocule analysis, headspace samples were helium-purged, N_2_O cryo-focused, purified, and analysed by GC-IRMS coupled with a Conflo IV interface (Delta V Plus, Thermo) to determine the 45/44 and 46/44 mass ratios, as well as the 31/30 ratio of the NO^+^ fragment. Calibration employed three N_2_O-in-air standards (CA06261, 53504, and CA08214), and corrections were applied using the *pyisotopomer* Python package, accounting for scrambling factors (6, 7). Site preference (SP = δ^15^Nα – δ^15^Nβ), defined by the distribution of the relative abundance of ^15^N in the central (α) and terminal (β) N atoms in N_2_O, was calculated to distinguish N_2_O production pathways, as SP is independent of δ^15^N and indicative of microbial and abiotic N_2_O sources (8 and references therein).

The isotopic signatures of NO_2_^-^, NO_3_^-^, and N_2_O reflect both substrate δ^15^N (i.e., NH_4_^+^) and the associated kinetic isotope effect (ε), which is defined as $\varepsilon= ((k_{heavy}/k_{light}) -1) x 1000$ and reported in parts per mille (‰), where $k$represents the rate constant for each isotope. The kinetic isotope effect describes the preferential use of lighter isotopes during biochemical transformations, leading to depletion of heavy isotopes in the product compared to the substrate. Under steady-state conditions in our reactor, the N isotope effects (^15^ε) values were estimated by the difference between the substrate (i.e., NH_4_^+^) and the downstream products (i.e., NO_2_^-^, NO_3_^-^, and N_2_O) (9).

Supplementary Figures


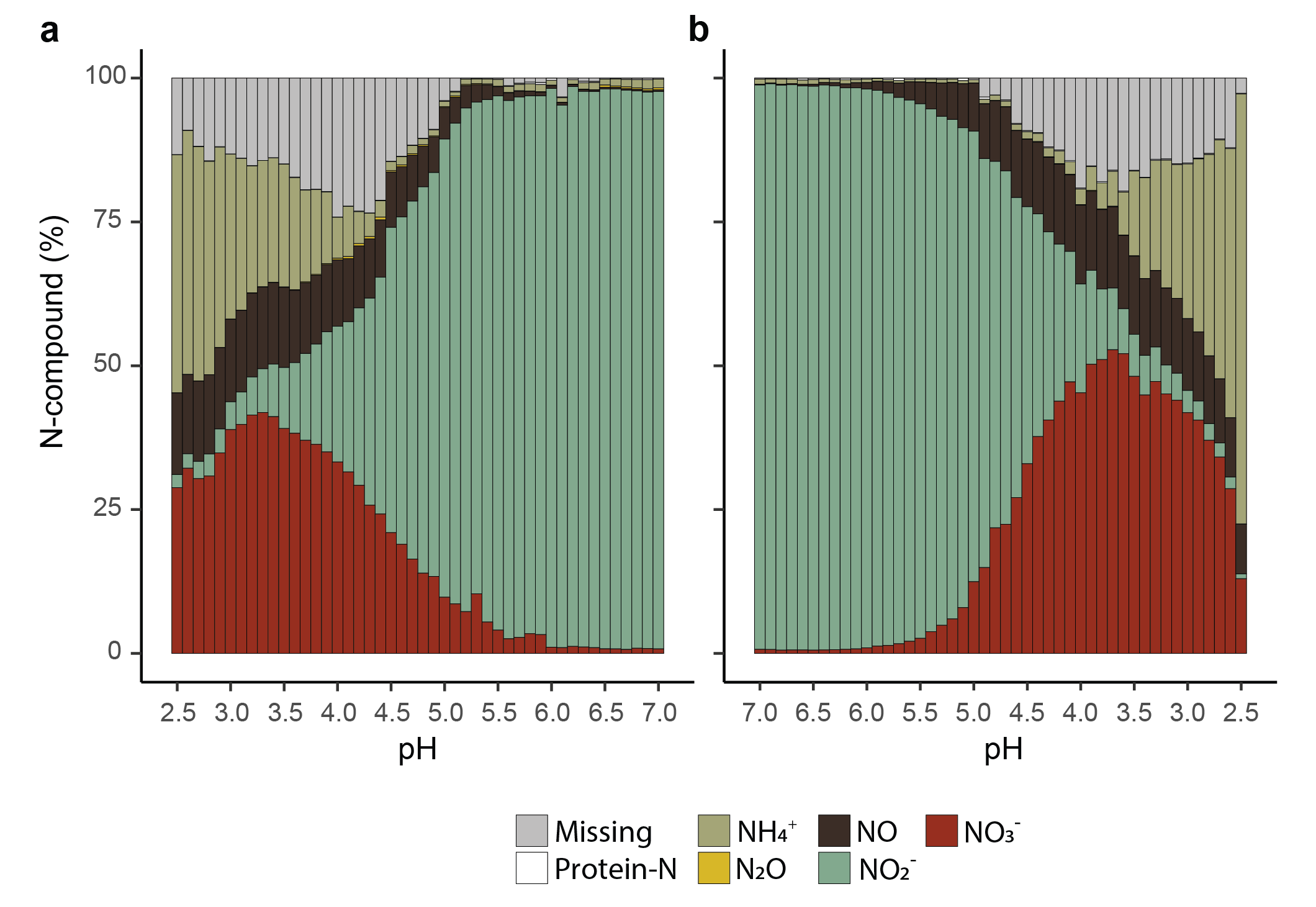


**Supplementary Figure 1** Nitrogen balances of “*Ca.* Na. tergens” sp. RJ19 enrichment bioreactor cultures fed with 70 mM NH_4_^+^. The relative amounts (in %) of residual NH_4_^+^ and the products NO_2_^-^, NO_3_^-^, NO, and N_2_O of the pH-up (**a**) and pH-down (**b**) bioreactor runs were included. All nitrogen inorganic compounds were measured; protein-N was estimated from the determined protein content, assuming a N-abundance of 16% (10). The difference to 100% of the supplied NH_4_^+^ was reported as “Missing”.


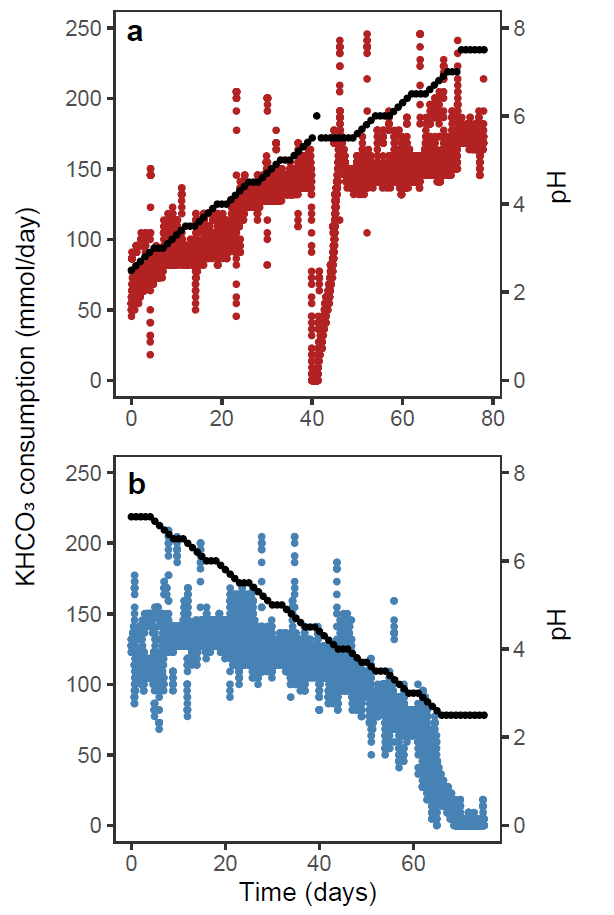


**Supplementary Figure 2** KHCO_3_ consumption of the bioreactor culture in mmol/day measured every 5 minutes continuously for the duration of the experiment as a proxy for the activity of the culture during (**a**) the pH-up (red dots) and (**b**) pH-down (blue dots) experiment. The black dots indicate the pH of the culture.


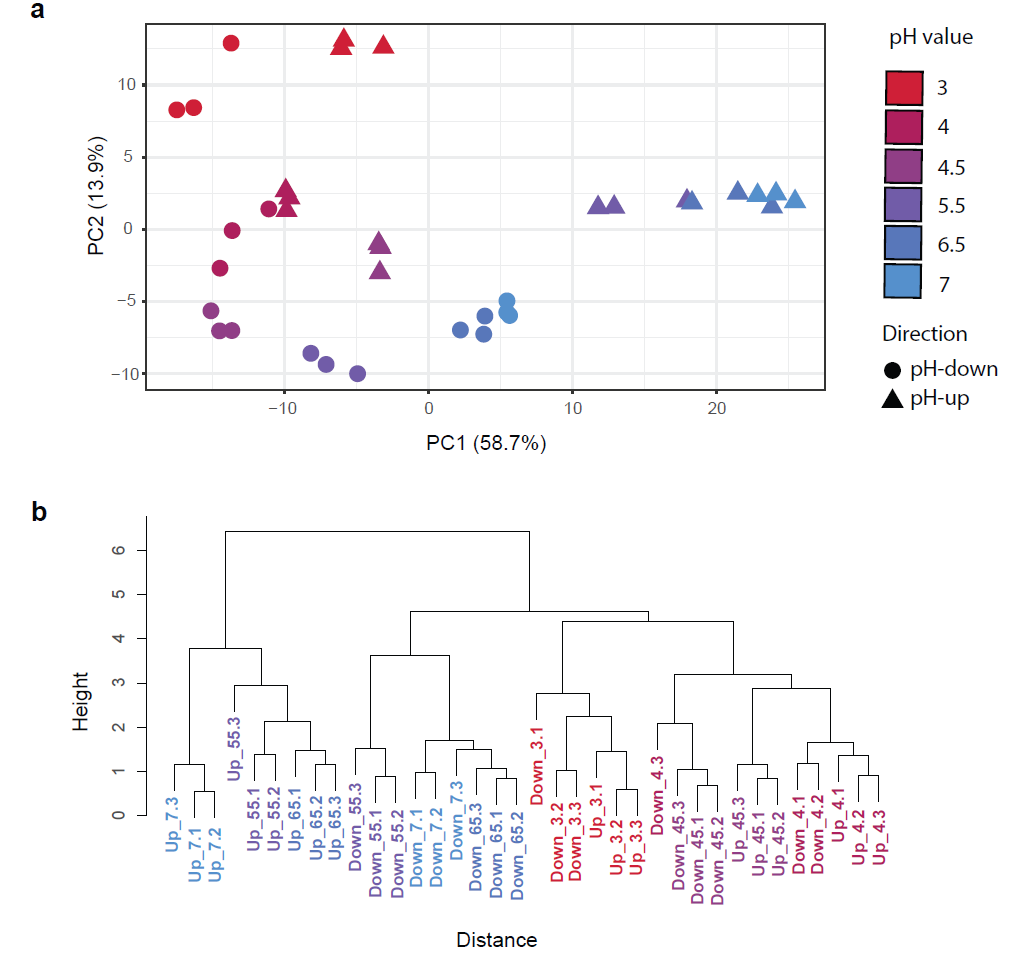


**Supplementary Figure 3** Principal component analysis of normalized gene expression profiles of “*Ca*. Na. tergens” (**a**) and a dendrogram illustrating the hierarchical relationships between the samples (**b**). pH conditions are indicated by a color gradient from red (most acidic) to blue (neutral pH). In (**b**), the pH-up and pH-down runs are indicated as “Up_” and “Down_”, respectively.

_
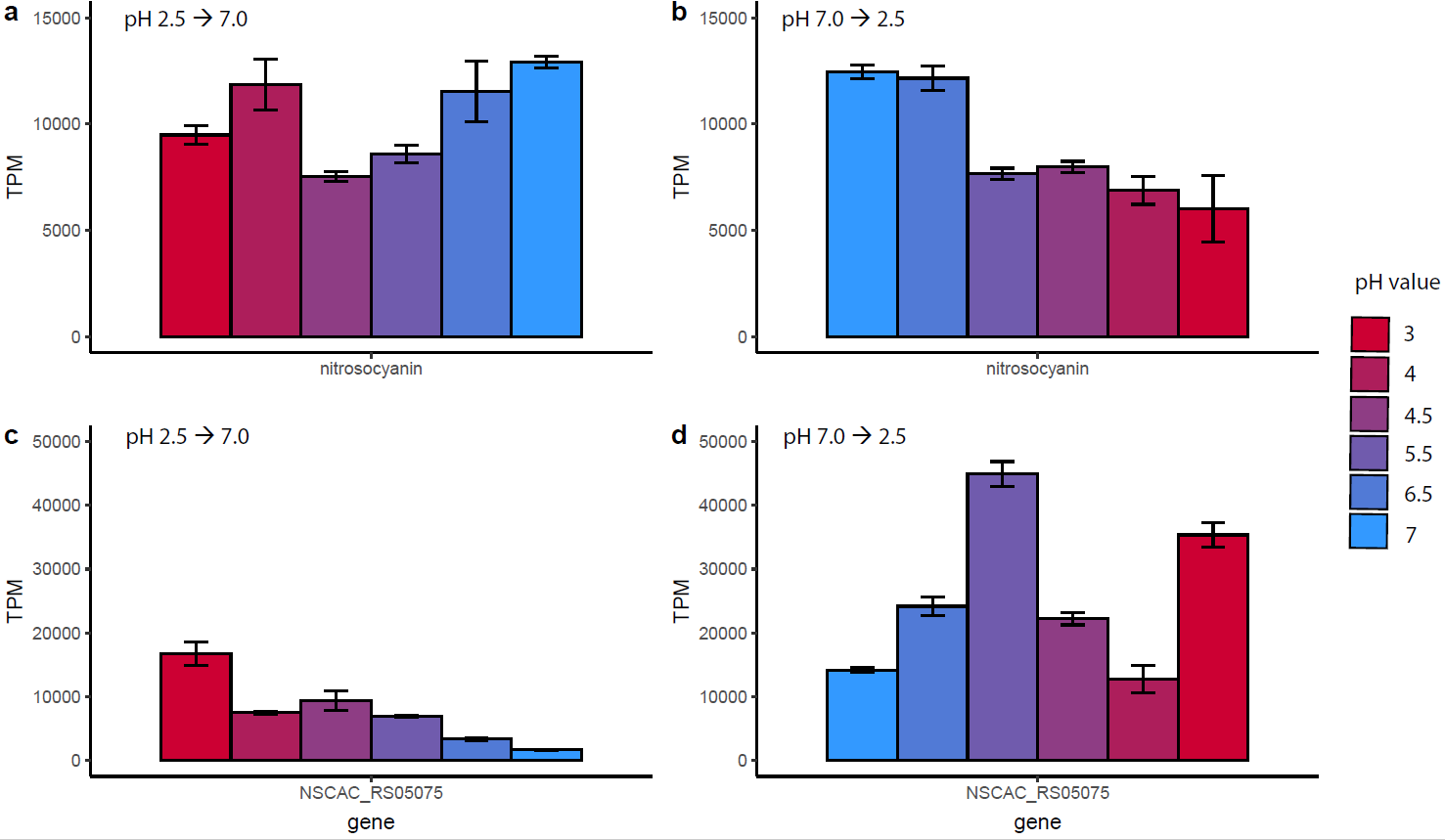
_

**Supplementary Figure 4** Transcripts per million of (**a, b**) nitrosocyanin and (**c, d**) the hypothetical protein NSCAC_1037 (RefSeq ID: NSCAC_RS05075) during the (**a, c**) pH-up and (**b, d**) pH-down experiments. Bars represent the average of triplicate transcriptomes, with pH indicated by a red (pH 3) to blue (pH 7) color gradient; error bars indicate the standard deviation.


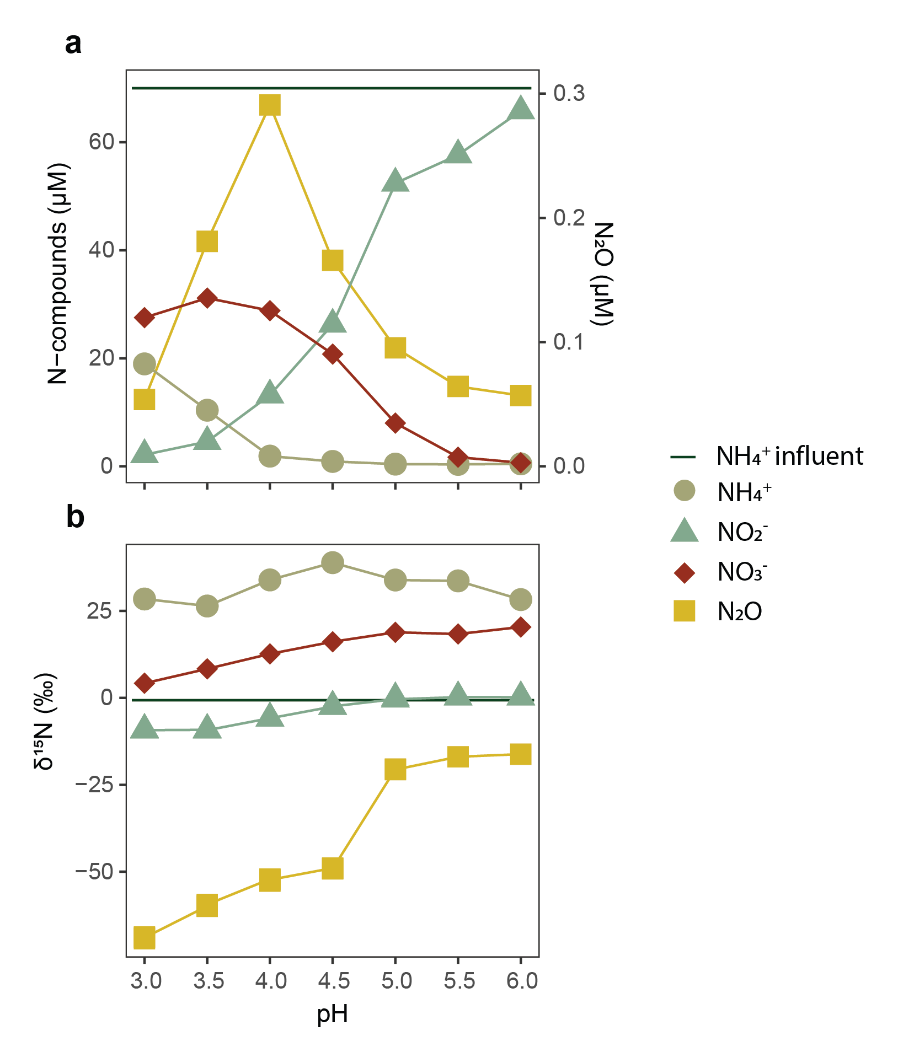


**Supplementary Figure 5** (**a**) Concentration of the N compounds NH_4_^+^ (olive green square), NO_2_^-^ (green triangle), NO_3_^-^ (red diamond), and N_2_O (yellow square) in the bioreactor culture between pH 6.0 and 3.0 during the pH-down experiment (in µM), and (**b**) the δ^15^N of these N compounds at these conditions (in per mille [‰]).

Supplementary Tables

**Supplementary Table S6** Summary of differentially expressed genes per pairwise comparison within the pH-up and pH-down runs. Cutoff values used were a log_2_-fold change ≥ 1 and an adjusted p value ≤ 0.01.

| **Direction** | **pH condition** | **Downregulated** | **Upregulated** | **Not Significant** |
| --- | --- | --- | --- | --- |
| pH-down | 6.5/7 | 0 | 1 | 1705 |
|  | 3/7 | 112 | 83 | 1511 |
|  | 5.5/6.5 | 2 | 20 | 1684 |
|  | 4/6.5 | 56 | 46 | 1604 |
|  | 4.5/5.5 | 3 | 1 | 1702 |
|  | 4/4.5 | 5 | 0 | 1701 |
|  | 3/4 | 27 | 7 | 1672 |
| pH-up | 4/3 | 5 | 53 | 1648 |
|  | 7/3 | 103 | 141 | 1462 |
|  | 4.5/4 | 2 | 10 | 1694 |
|  | 6.5/4 | 143 | 144 | 1419 |
|  | 5.5/4.5 | 52 | 33 | 1621 |
|  | 6.5/5.5 | 10 | 14 | 1682 |
|  | 7/6.5 | 4 | 2 | 1700 |

**References**

1. McIlvin MR, Altabet MA. 2005. Chemical conversion of nitrate and nitrite to nitrous oxide for nitrogen and oxygen isotopic analysis in freshwater and seawater. Anal Chem 77:5589-5595.

2. García-Robledo E, Corzo A, Papaspyrou S. 2014. A fast and direct spectrophotometric method for the sequential determination of nitrate and nitrite at low concentrations in small volumes. Mar Chem 162:30-36.

3. Zhang L, Altabet MA, Wu T, Hadas O. 2007. Sensitive measurement of NH4+ 15N/14N (δ15NH4+) at natural abundance levels in fresh and saltwaters. Anal Chem 79:5297-5303.

4. Sigman DM, Casciotti KL, Andreani M, Barford C, Galanter M, Böhlke J. 2001. A bacterial method for the nitrogen isotopic analysis of nitrate in seawater and freshwater. Anal Chem 73:4145-4153.

5. Casciotti KL, Sigman DM, Hastings MG, Böhlke J, Hilkert A. 2002. Measurement of the oxygen isotopic composition of nitrate in seawater and freshwater using the denitrifier method. Anal Chem 74:4905-4912.

6. Frame CH, Casciotti K. 2010. Biogeochemical controls and isotopic signatures of nitrous oxide production by a marine ammonia-oxidizing bacterium. Biogeosciences 7:2695-2709.

7. Kelly CL, Manning C, Frey C, Kaiser J, Gluschankoff N, Casciotti KL. 2023. Pyisotopomer: A Python package for obtaining intramolecular isotope ratio differences from mass spectrometric analysis of nitrous oxide isotopocules. Rapid Commun Mass Spectrom 37:e9513.

8. Yu L, Harris E, Lewicka‐Szczebak D, Barthel M, Blomberg MR, Harris SJ, Johnson MS, Lehmann MF, Liisberg J, Müller C. 2020. What can we learn from N2O isotope data?–Analytics, processes and modelling. Rapid Commun Mass Spectrom 34:e8858.

9. Sigman DM, Casciotti K. 2001. Nitrogen isotopes in the ocean, vol 3.

10. Mariotti F, Tomé D, Mirand PP. 2008. Converting nitrogen into protein—beyond 6.25 and Jones' factors. Crit Rev Food Sci Nutr 48:177-184.
